# A MRS study of metabolic alterations in the frontal white matter of major depressive disorder patients with the treatment of SSRIs

**DOI:** 10.1186/s12888-015-0489-7

**Published:** 2015-05-02

**Authors:** Yifan Zhang, Yu Han, Yongzhi Wang, Yinfeng Zhang, Li Li, Erhu Jin, Ligang Deng, Brandi Watts, Teresa Golden, Ning Wu

**Affiliations:** 1 Department of Traditional Chinese Medicine, Beijing Friendship Hospital, Capital Medical University, 95 Yong-An Road, Beijing, 100050 China; 2 Department of Radiology, Beijing Friendship Hospital, Capital Medical University, Beijing, 100050 China; 3 Department of Biological Sciences, Southeastern Oklahoma State University, Durant, OK 74701 USA

**Keywords:** Antidepressant, Magnetic resonance spectroscopy, Major depressive disorder, Brain metabolism

## Abstract

**Background:**

Proton magnetic resonance spectroscopy provides a non-invasive technology to study brain metabolite levels *in vivo*, which can be used to measure biochemical compounds or metabolite concentrations in circumscribed brain regions. Previous research has highlighted the role of glial cells in brain white matter. It has been assumed that antidepressant treatment with SSRIs not only affects neurons, but also activates glial cells. This study focused on the observation of any potential changes in the metabolite levels of the ventral prefrontal white matter in major depressive disorder (MDD) patients who have received antidepressant treatment.

**Methods:**

17 female patients diagnosed as MDD according to Diagnostic and Statistical Manual of Mental Disorders, Fourth Edition (DSM-IV) criteria with the scores of 18 and above on the 24-item Hamilton Depression Rating Scale (HDRS) were recruited. MRS studies were performed on a 3.0 T MR system, single voxel PRESS spectroscopy with chemical-shift selective saturation water suppression. The volume of interest was localized at the bilateral ventral prefrontal white matter regions (voxel size: 2 × 2 × 2 mm^3^). The spectral data analysis was performed by using the instrument manufacturer supplied software.

**Results:**

The bilateral ventral prefrontal white matter of MDD patients showed significantly lower Cho/Cr (p < 0.05) before receiving treatment. The HDRS, as the indicator of treatment response, showed a significant decrease in patients who had gone through 12 weeks treatment (p < 0.01). The bilateral Cho/Cr values of post-treatment patients were increased significantly compared to that of pre-treatment (p < 0.05).

**Conclusion:**

The alteration of ventral prefrontal white matter metabolite levels are likely involved in MDD pathophysiology and imply a crucial role of white matter in MDD.

## Background

Major depressive disorder (MDD) is a highly prevalent psychiatric disorder. Major symptoms of this disease are characterized by persistent feelings of depressed mood, loss of motivation, feelings of worthlessness and suicidal tendencies. The understanding of the nature and causes of depression has evolved over the centuries, although this understanding is incomplete and has left many aspects of depression as the subject of discussion and research. Proposed causes include psychological, psychosocial, hereditary, evolutionary and biological factors [1]. Although brain abnormalities have been found in MDD patients by numerous neuroimaging studies, the precise pathophysiological mechanisms of depressive disorder are still not clear.

The main method for antidepressant treatment is pharmacological. Selective serotonin reuptake inhibitors (SSRIs) are widely used in the treatment of depression. The diagnosis of MDD is mainly based on clinical signs and symptoms, and treatment protocols are established based on clinical empirical evidence [2,3]. Exploration of neurological biomarkers for diagnosis and treatment of MDD such as proton magnetic resonance spectroscopy has the potential to predict the response to treatment in patients with MDD.

Proton magnetic resonance spectroscopy (1H-MRS) is a non-invasive MRI technique that can quantify the concentration of multiple metabolites, including N-acetyl-aspartate (NAA), choline (Cho), and creatine (Cr). NAA is generally known as a marker of neuronal density and viability, as lower concentrations of this metabolite are thought to be indicative of loss of neural function [4,5]. Cho is a considered to be the marker of membrane integrity and altered levels of this molecule are also associated with neurobiological diseases. Cr reflects ATP metabolism and production and is relatively constant across the brain. It is generally used as a reference metabolite to which other metabolites are normalized to [6,7].

Reviews and meta-analyses analysis of the 1H-MRS literature on major depression found evidence that abnormalities in the hippocampus, basal ganglia and prefrontal lobes were present. Evidence indicates a correlation between changes in neuro-metabolite concentrations, in particular glutamate, NAA, GABA and choline, with a positive treatment response to pharmacotherapy or antidepressant stimulation techniques [8]. In earlier MRS studies of MDD, Sonawalla et al. found that Cho/Cr ratios increased after 8 weeks of fluoxetine treatment in the basal ganglia [9]. Gonul et al. found decreased NAA/Cr ratios in depressive patients and these levels in the left medial frontal cortex may increase significantly after treatment with SSRIs [10]. Alternatively, there are also MRS studies in depression that reported positive treatment responses to sleep deprivation (SD), electroconvulsive therapy (ECT), or repetitive transcranial magnetic stimulation (rTMS) in the DLPFC, amygdala, pontine, hippocampus, and the occipital cortex [11-16]. However, up until now, evidence for the effects of antidepressive treatment with SSRIs on frontal white matter regions in humans has rarely been reported.

The prefrontal areas play an important role in mood regulation. This includes the ventral prefrontal lobe including the medial PFC, orbitofrontal cortex, subgenual PFC, a part of ACC, and white matter structures. Proper function of these regions is involved in the coupling of thoughts, memories, and experience with corresponding emotional and visceral states. These regions are often referred to as “paralimbic” regions and play an important role in linking cognition with visceral states and emotion [17,18]. The prefrontal lobe cortex can pass the neural signals to the limbic system including the regions of cingulate gyrus, amygdala, etc. through the white matter neural fibers, which composes the MDD neural emotional signal transduction pathway. Therefore, the ventral prefrontal white matter is the major transduction pathway that connects the cortex of the prefrontal lobe and the regions of limbic system. Earlier studies of SSRIs focused on neuron and neurotransmission systems and seldom do these articles address to the role of white matter [19].

A recent study however has highlighted the role of glial cells in the brain frontal white matter. It had been assumed that antidepressant treatment not only affects neurons, but also activates glial cells [20]. Previous articles on MDD indicate that studies using Structural MRI, fMRI, diffusion tensor imaging (DTI) and Postmortem show abnormalities or impairments in ventral prefrontal white matter [21-24]. These studies may suggest that prefrontal white matter plays an important role in the pathophysiology of MDD. Therefore, the purpose of our study was to observe the biochemical alterations in ventral prefrontal white matter and examine whether there was any effect of antidepressant treatment on the metabolite levels in this area in the depressed patients.

## Methods

### Subjects

The protocol was approved by the ethics committee of the Beijing Friendship Hospital and the studies were carried out in strict accordance with the Declaration of Helsinki. All participants were female and right-handed. 17 patients (mean age 43 ± 10 years) diagnosed as MDD according to Structured Clinical Interview for the Diagnostic and Statistical Manual of Mental Disorders, Fourth Edition (DSM-IV), with scores of 18 or greater on the 24-item Hamilton Depression Rating Scale (HDRS) were recruited for the study group. In addition, 19 healthy volunteers (mean age 41 ± 10 years) were recruited as normal control. All normal subjects were carefully screened by a diagnostic interview, a Structured Clinical Interview for DSM-IV Non-patient Edition, to rule out the presence of current or past psychiatric illness. All participants signed an “Informed Consent” form after receiving a complete description of the study. All patients had not taken any antidepressants for at least 8 weeks prior to the examination and had the duration of illness over 24 months. The following exclusion criteria was applied to all participants: (1) the presence of other psychiatric disorders and symptoms, (2) a history of treatment with any psychotropic medication, psychotherapy or electroconvulsive therapy, (3) a history of neurological or organic brain disorder, (4) alcohol/substance abuse within 6 months before study entry, (5) any physical illness demonstrated by personal history, or clinical or laboratory examinations, and (6) first-degree relatives showing a history of neurological or mental illness.

### MRS scanning procedure

MRS scanning was performed on both control and patient groups before SSRIs treatment. The patient group received the second MRS examination about 12 weeks after applying SSRIs treatment (Paroxetine, 20 mg/d). Depression severity at follow-up was also evaluated by using the HDRS.

**Figure 1:**
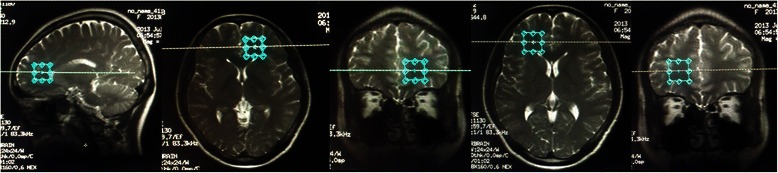
Sagittal, coronal and axial scout MR images showing an 8-cm^3^ voxel predominantly centered white matter of the bilateral ventral prefrontal lobe.

MRS studies were performed on a 3.0 T MR system (General Electric, Excite Signa HD 3.0 T). A standard eight-channel head coil was used for radio frequency transmission and reception of the MR signal. Magnetic resonance imaging examination protocol included three dimensional fast-spoiled gradient-echo (3D-FSPGR) sequence [repetition time (TR)/echo-time (TE) = 6/2.5 ms, slice thickness: 1 mm, number of slices: 160, interslice gap: 0 mm, field of view (FOV): 220 × 220, number of excitation (NEX) = 1] that were obtained to confirm the absence of any structural and signal abnormality of the brain. Single voxel PRESS (spin-echo point resolved) spectroscopy (TR/TE: 1500/35 ms, voxel size: 2 × 2 × 2 mm^3^, field of view: 24 × 24,number of excitation = 8) with chemical-shift selective saturation (CHESS) water suppression was used for 1H MR Spectra measurements. The volume of interest (VOI) was localized at the bilateral ventral prefrontal white matter regions. According to the mechanical requirements of GE 3.0 T MR system, the area of VOI was set as 2 × 2 × 2 = 8 cm^3^. The VOI boarders were determined by avoiding any potential cerebral spinal fluid area and including as much as possible of the white matter tissue regions. Figure 1 shows the VOI location in brain MRI scanning. During voxel placement, avoiding the adjacent grey matter containing some part of the prefrontal was not achieved because of the voxel size. Total acquisition time for 1H MRS sequence was 4 min and 36 s. The analysis of the spectral data was performed with the MRI manufacturer-supplied software (GE Advantage Workstation: AW4.2). The values of the NAA/Cr, Cho/Cr ratios were automatically analyzed by the MR system. Voxel placements for spectroscopy and all data analysis was carried out by a trained radiologist who was blind to each subject’s diagnosis.

### Statistical analysis

All data analysis was performed by using SPSS for Windows software, version 13.0 (SPSS Inc. Chicago, IL, USA) and two-tailed significance level was set at 0.05. Independent sample *t*-test was used to compare demographic data between the MDD and healthy control groups at pre-treatment condition. To assess treatment response, Hamilton Depression Scale and 1H-MRS measures were compared between pre-treatment and post-treatment of patients by paired t tests. Data was presented as means and standard deviations.

## Results

The clinical symptoms of all participating patients were significantly improved after the completion of the SSRI treatment course. Both HAM-A and HAM-D scores of each individual patient showed significant reduction after the SSRI treatment compared to that before the treatment (data not shown).

### Pre-treatment MRS comparison

The pre-treatment comparison study between MDD patients and normal control groups revealed that there was significantly lower Cho/Cr in bilateral ventral prefrontal white matter of MDD patients than in the normal control group (both left and right sides: p < 0.05). However, there was no significant difference on NAA/Cr levels in bilateral ventral prefrontal white matter between the two groups. Table 1 summarizes the results of Independent sample *t*-tests with mean value (SD) for relative metabolite concentrations in the bilateral ventral prefrontal white matter volume of interest.

**Table 1:** **Pre-treatment MRS exam of patient and control groups**

	Left frontal white matter regions		Right frontal white matter regions	
	NAA/Cr	Cho/Cr	NAA/Cr	Cho/Cr
Patient (n = 17)	1.51 (0.17)	0.99 (0.09)	1.61 (0.16)	0.97 (0.11)
Controls (n = 19)	1.59 (0.15)	1.08 (0.11)	1.63 (0.13)	1.10 (0.15)
t value	0.91	2.45	0.31	2.53
P value	0.31	0.03	0.20	0.04

### Post-treatment MRS comparison

**Figure 2:**
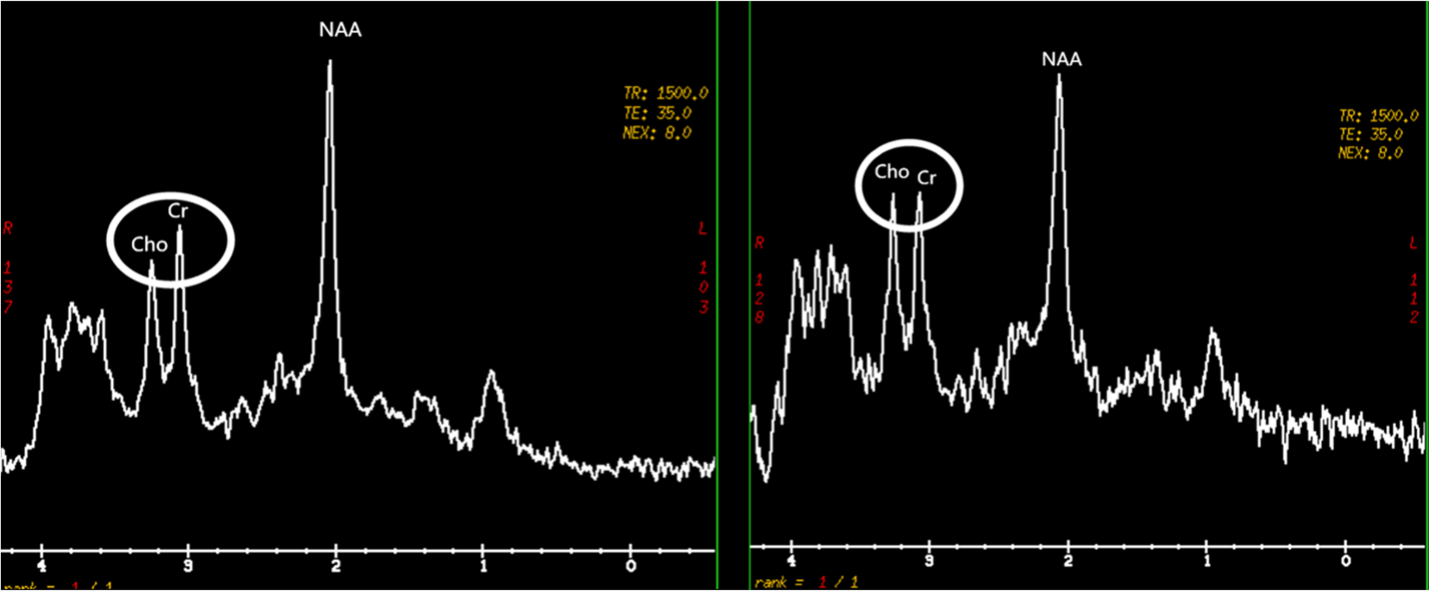
Comparison of the same patient’s pre- (left) and post- (right) treatment Cho/Cr level.

After applying SSRI treatment (Paroxetine, 20 mg/d) for 12 weeks, the HDRS had significantly decreased in MDD patients (p < 0.01), which indicated a positive response to the treatment. All patients’ second MRS exam results demonstrated significant increases of bilateral Cho/Cr values compared to the same patient’s pre-treatment value (Figure 2). Paired sample *t*-test results showed that there was significant difference between the pre- and post-treatment in bilateral ventral prefrontal white matter of the same patient (left side: p < 0.01; right side: p < 0.05) (Table 2). However, there was still no significant change on NAA/Cr level in bilateral ventral prefrontal white matter after treatment. Results of metabolite ratio comparison are summarized in Table 3.

**Table 2:** **Pre- and post-treatment comparison of patients’ Cho/Cr ratio in bilateral ventral prefrontal white matter**

	Left Cho/Cr		Right Cho/Cr	
	Pre-treatment	Post-treatment	Pre-treatment	Post-treatment
1	0.83	0.92	0.99	1.11
2	0.91	0.92	0.95	1.09
3	0.91	0.98	0.80	1.01
4	0.93	0.98	0.98	1.05
5	0.93	1.12	1.04	1.19
6	0.95	1.00	0.86	1.04
7	0.96	0.98	1.01	0.95
8	0.99	1.04	1.12	1.09
9	0.99	1.05	1.14	1.15
10	1.02	1.04	1.04	1.05
11	1.03	1.13	0.97	1.04
12	1.03	0.98	1.15	1.08
13	1.03	1.07	0.8	0.87
14	1.06	1.03	1.16	1.01
15	1.07	1.11	0.97	1.04
16	1.12	1.13	0.49	0.95
17	1.23	1.22	0.99	1.01

**Table 3:** **Post-treatment MRS exam of patient group**

	Left frontal white matter regions		Right frontal white matter regions	
	NAA/Cr	Cho/Cr	NAA/Cr	Cho/Cr
Pre-treatment	1.51 (0.17)	0.99 (0.09)	1.61 (0.16)	0.97 (0.11)
Post-treatment	1.51 (0.12)	1.05 (0.07)	1.60 (0.13)	1.04 (0.08)
t value	0.63	−3.13	0.266	−2.24
P value	0.53	0.006	0.79	0.04

## Discussion

To our knowledge, this is the first study using MRS to detect a drug response in the bilateral ventral prefrontal white matter in patients with MDD.

Two major metabolite ratios (NAA/Cr and Cho/CR) were evaluated in this study through comparative analysis of patient/control pre-treatment MRS data and the same patient pre-/post- treatment MRS results. There were no significant differences found on NAA/Cr level in both pre- and post-treatment results. The NAA functions in the brain as an acetyl donor for acetyl coenzyme A and has been implicated in several neuronal processes, including lipid and protein synthesis, mitochondrial functioning, and osmoregulation [25,26]. NAA is commonly considered to be a marker of neuronal and axonal integrity [27,28]. Thus, reduction in NAA concentration possibly reflects the loss of neural function. Our results indicated that the NAA levels showed no difference between the patient and control groups. A possible explanation for such results is due to the restricted selection of VOI used in this study. The selected area was mainly focused on prefrontal white matter in which glial cells accounted for the majority while the primary site for NAA synthesis is in the neurons of the cortex [25].

The results did show a significant reduction of Cho/Cr level in the ventral prefrontal white matter of MDD patients compared to that of controls. Cho is considered to be a potential biomarker for the status of membrane phospholipid metabolism [29]. Reduction of Cho levels has been associated with decreased membrane turnover and/or impaired intracellular signal transduction systems [30,31]. Alternatively, as Cho is hugely present in glia cells and myelin [32], the lower ratio of Cho/Cr may indicate that abnormalities exist in membrane structure and function of glial cells, and myelin. Previous morphometric MRI has shown volumetric reductions in the ventral prefrontal regions. Diffusion Tensor Imaging (DTI) studies found decreased white matter integrity in different white matter tracts in MDD [33]. Postmortem studies also reported lower density of oligodendroglial cells in the prefrontal region of patients with MDD [34]. Taken together, our results suggest reduced bilateral Cho/Cr in patients in the ventral prefrontal white matter indicated that the prefrontal white matter may also play a role in the pathophysiology of MDD. By thoroughly reviewing the literature and meta-analysis results of the 1H-MRS on major depression through 2012, there was no consistent evidence found that NAA and Cho are either increased or decreased in patients with major depression. This appears to be evidence that brain chemistry varies across different regions and thus warrants further investigation.

The post-treatment MRS results suggested that patients’ Cho/Cr values in bilateral prefrontal white matter were significantly increased compared to the pre-treatment values. White matter structure consists mostly of glial cells and myelinated axons that transmit signals from one region of the cerebrum to another. Previous studies of SSRI treatment had little focus on the role of glial cells. The glial cells were only considered to actively participate neuronal network activity. Their passive supportive role of neuronal functions were mostly highlighted. Recent studies suggest that glia cells are divided into three types, including astrocytes, oligodendrocytes, and microglia [20,35]. Astrocytes contribute to regulation of neurotransmission through their processes wrapping around synapses and to modulation of brain blood flow of the blood–brain-barrier and brain blood flow through their end-feet surrounding blood vessels. Oligodendrocytes are important in forming the myelin sheaths around axons that guarantee conduction of electrical stimuli for long distances without increasing axonal diameters. Microglia cells represent the resident CNS immune cells and are fundamental surveyors of CNS extracellular environment that may help the to maintain or restore homeostasis through pruning of inappropriate synaptic contacts. Metabolic alterations of frontal white matter after treatment with SSRIs in our study may indicate a strong connection with this role in the function of these glial cells.

The previous animal experiments found that antidepressant therapies acted on glial cells. Studies demonstrated that mammalian myelin-forming cells require the expression of some glia-specific proteins and genes to maintain neuronal and axonal integrity [36-38]. There is evidence that antidepressant treatments have a profound stimulatory effect on the expression levels of various trophic factors. It is also reported that antidepressant actions of SSRIs could modify astroglial physiology and morphology by affecting gliogenesis. The antidepressants may even regulate glial cell numbers [20,39]. The results of animal experimental studies indicate that SSRIs may not only affect neurons but also activate glial cells. However, the underlying molecular mechanisms are still unclear. Our results demonstrated that bilateral Cho/Cr of patients in white matter were increased with SSRI treatment and indicated that SSRIs might have a positive effect on the regulation of glial cells and axons. Previous MRS studies in depression reported abnormalities in the frontal cortex, basal ganglia, hippocampus, anterior cingulate cortex, and the occipital cortex. These abnormalities were improved after treatment with selective serotonin reuptake inhibitor, electroconvulsive therapy, and other antidepressant therapy. These results indicated that antidepressant treatment with its neurotrophic and neuroplasticity effects might play a positive role in restoring neuronal and glial integrity.

Glial cells are active partners of neurons that regulate the arrangement of neuronal circuits in specific brain regions. Our results indicate that glial cells might be involved with antidepressant action in restructuring neuronal and glial integrity. The results of this study may be useful to improve current treatment regimens or identify novel targets for the development of more efficacious antidepressant drugs.

## Conclusion

This study is the first one conducted to evaluate SSRI drug response in the bilateral ventral prefrontal white matter in patients with MDD using MRS technology. The results suggest that alterations in ventral prefrontal white matter metabolite levels are likely to be involved in MDD pathophysiology and may help in understanding the pathophysiology and the crucial role of white matter in MDD.

## Abbreviations

Cho: Choline
Cr: Creatine
Cr + PCr: Creatine/phosphocreatine
DLPFC: Dorsolateral prefrontal cortex
HAM-D: Hamilton depression rating scale
HAM-A: Hamilton anxiety rating scale
MRS: Proton magnetic resonance spectroscopy
GABA: Gamma-aminobutyric acid
PFC: Prefrontal cortex
ACC: Anterior cingulate cortex
PRESS: Point resolved spectroscopy
CNS: Central nervous system
